# The *APOB* rs693 polymorphism impacts the lipid profile of Brazilian older adults

**DOI:** 10.1590/1414-431X20199102

**Published:** 2020-03-02

**Authors:** E.S. Alves, A.D. Henriques, A.C. Tonet-Furioso, R.S. Paula, L.O. Gomes, C.F. Moraes, O.T. Nóbrega

**Affiliations:** 1 Programa de Pós-Graduação em Ciências da Saúde, Faculdade de Ciências da Saúde, Universidade de Brasília, Brasília, DF, Brasil; 2 Programa de Pós-Graduação em Ciências Médicas, Faculdade de Medicina, Universidade de Brasília, Brasília, DF, Brasil; 3 Programa de Pós-Graduação em Gerontologia, Escola de Saúde e Medicina, Universidade Católica de Brasília, Brasília, DF, Brasil; 4 Research Centre, Institut Universitaire de Gériatrie de Montréal, Montreal, Quebec, Canada

**Keywords:** Apolipoprotein B, Genetic polymorphism, Elderly, Hyperlipidemia, Diet

## Abstract

The apolipoprotein B (*APOB*) gene contains several polymorphic sites described as risk modifiers for cardiovascular events. The objective of this study was to verify the association of the classic *APOB Xba* I polymorphism (rs693) with atherosclerotic risk factors in a segment of the Brazilian elderly population considering their usual dietary intake. Clinical and biochemical characteristics as well as total caloric and fat intake data were determined from 644 elderly individuals. Polymorphism analysis was performed by conventional polymerase chain reaction followed by enzyme restriction. Statistical analyses compared measures and proportions according to different *APOB* genotypic combinations. Statistically significant association was found between *Xba* I polymorphism and serum LDL, total cholesterol, and total lipid levels, with important elevations among T homozygotes compared to the other genotypes. There was homogeneity in all other parameters analyzed (including intake pattern), with a tendency for reduced levels of circulating apolipoprotein B among TT individuals. Our results pointed out that genetic variation in *APOB* affected the lipemic profile of elderly individuals in a context not biased by diet, generating a pattern suggestive of secretory disorder of lipoprotein particles, with possible implication in atherosclerotic risk.

## Introduction

With aging, numerous physiological changes occur and the risk for chronic diseases increases (1). Cardiovascular disease (CVD) is one of the leading causes of morbidity and mortality worldwide, accounting for about 31% of all deaths in 2016 (2). The hallmark to these diseases is atherosclerosis, a chronic low-grade inflammatory phenomenon in cholesterol-enriched vascular beds that is modulated by modifiable (obesity, smoking, hypertension, dyslipidemia, diabetes) as well as non-modifiable (age, gender, and genetic factors) contributors for its onset (3).

The literature consistently reassures the association between dyslipidemia and incidence of vascular events, with strong etiological contribution by increased triglyceride (TG), low-density lipoprotein (LDL), very-low-density lipoprotein (VLDL), and apolipoprotein B (apoB) levels, as well as by reduction of high-density lipoprotein (HDL) levels (4,5). With subendothelial retention of apolipoprotein(apo)B-containing particles as a necessary trigger for atherogenesis, levels of this apolipoprotein present predictive power for vascular events as high as those of LDL (6,7).

As cardiovascular risk markers, lipoprotein concentrations are influenced by environmental conditions such as diet and lifestyle, and allelic configurations incidentally contribute to this association (8). Genome-wide association studies have identified several candidate *loci* for susceptibility to dyslipidemia and CVD, including apolipoprotein genes (4,9). Considering that apoB is a key structural protein in atherogenic metabolites as chylomicrons, VLDL, and LDL, and as the main ligand to hepatic LDL receptors, the remarkably wide locus of the *APOB* gene (ENSG00000084674) at 2p23-24 contains several polymorphic sites described as modifiers of the risk of cardiovascular events (9,10).

In this context, epidemiological studies have demonstrated association of gene polymorphisms both with serum elevation of total cholesterol (TC) and lipid subfractions (mainly LDL and TG), as well as with the development of atherosclerosis, with emphasis on rs693 single nucleotide polymorphism (SNP), also known as the *Xba* I variation, which consists of a silent transition (ACC → ACT) in exon 26 (11). In a case-control study, patients carrying the T allele presented an average 2-fold higher risk of dyslipidemia compared to controls, while homozygotes exhibited a 4-fold increased risk (12). A higher prevalence of atherosclerotic plaques in carotid arteries of T homozygotes has also been reported (13). Some studies have also found association of this polymorphism with CVD, increased apoB, TC, TG, and LDL levels, and decreased HDL levels (5,14–17). On the other hand, some studies found no association between these parameters (9,11,18).

Although the influence of apoB gene and its *Xba* I polymorphism on interindividual variability in lipemic levels and cardiovascular risk has been extensively investigated, the results of studies are inconsistent and conflicting regarding the main parameters affected, appearing to differ according to diet and population group studied. Thus, this study aimed to verify the association of *Xba* I polymorphism (rs693) of the apolipoprotein B gene with atherosclerotic risk factors, including lipemic profile, in a segment of the Brazilian elderly population.

## Material and Methods

### Sample

The study sample included 644 non-institutionalized and unrelated patients aged 60 years or older recruited from geriatric outpatient clinics located in the Federal District, Brazil, namely: Geriatric Service of the Catholic University Hospital of Brasília (HUCB) and the Multidisciplinary Center for the Elderly of the University Hospital of Brasília (HUB/UnB). Inclusion criterion was the spontaneous search for primary or secondary care for circulatory events. Exclusion criteria were: active and/or infectious inflammatory condition, neoplasia of any type (current or past), major renal insufficiency (creatinine clearance <25 mL·min^-1^·(1.73 m^2^)^-1^, with or without abnormalities in the liver function marker levels. No active search for patients with any specific condition was performed.

The equation reproduced by Whitley and Ball (19) was used to calculate the study power to detect minimum change of 10 mg/dL in LDL levels. Considering that the overall standard deviation of the variable was of the order of 30 mg/dL, the standardized difference to be detected was 10/30=0.333. Thus, 425 participants were required to detect such difference, with a power of 80% and cutoff point of 0.05 as threshold of statistical significance, which makes the study sufficiently capable of detecting differences.

The study was approved by the Ethics Research Committee of Faculty of Medicine of the University of Brasília, and all participants signed the free consent form before the beginning of data collection, granting express authorization for the constitution of a biobank for present and subsequent use of data and biological samples. In addition, the project was registered at the National System of Management of the Genetic Heritage and Traditional Associated Knowledge (SisGen), under access A407626.

### Clinical evaluation

Clinical evaluation of patients was performed at both outpatient clinics following a standardized protocol. Systolic and diastolic blood pressure values were measured according to recommendations of the VI Brazilian Guideline for Hypertension (20). Data regarding the use of drugs to control dyslipidemia were also collected. In the anthropometric analysis, body mass index (BMI) and body fat were evaluated. Patients were weighed in light clothing and without shoes. Height was measured with a stadiometer fixed on the wall, with the patient standing on a firm level surface with arms along the body. BMI was obtained by dividing weight (in kg) by the squared height (in m), while body fat was defined by dual energy X-ray absorptiometry (DXA, model Lunar DPX-IQ, software version 4.7e, Lunar Radiation Corp, USA) performed according to manufacturer's recommendations, with absolute fat (kg) converted into fat (%) relative to body weight.

Patients were evaluated with the Portuguese-validated version of the Mini-Mental State Exam (22). To increase data reliability on dietary records, patients with a score <11/30 among the illiterate, <17/30 among those with up to 7 years of formal education, and <25/30 among those with 8 years of formal education were excluded from our analysis (23).

### Biochemical analyses

Biochemical analyses included determination of blood glucose, TC, LDL cholesterol, VLDL cholesterol, HDL cholesterol, triglycerides, total lipids (TL), C-reactive protein (CRP), apolipoprotein B (apoB), creatinine clearance, and thyroid stimulating hormone (TSH). Venous blood samples were collected in tubes with clot activator after fasting for up to 12 h, and the material was centrifuged at 2000 *g* for 15 min at 5°C for serum separation. Tests were performed following routine clinical analyses with reagents from Boehringer Mannheim (Germany), and processed on AutoHumalyzer (Human GMBH, Germany) or nephelometer equipment (DadeBehring, Germany). The Friedewald equation was used to produce LDL cholesterol estimates (21).

### Diet evaluation

Total energy consumption was determined by 3-day food registry (2 days of the weekdays and 1 day of the weekend), filled at home after instructions from a clinical nutritionist to register number and size of food servings. Individual ingestion values are reported as mean values ingested over the three days of food registry. Forms were returned during clinical interview in which quantities and types of food were reviewed, and missing data was obtained and added. The caloric content of each diet was calculated using the Diet-Pro^®^ software, version 4.0 (A.S. Sistemas, Brazil), configured for international food tables and supplemented with a table for local foods (24,25). Also, dietary intake of total lipids as well as of saturated, monounsaturated, and polyunsaturated fatty acids (abbreviated SFA, MUFA and PUFA, respectively) are reported in terms of percentage relative to that of total energy.

### DNA extraction and *APOB* genotyping

Each participant in the study was submitted to blood collection for freezing on the occasion of biochemical analyses. DNA extraction used standard kits (QIAamp DNA Mini Kit, Qiagen, Brazil), according to manufacturer's recommendations. For the identification of genotypes, a segment of the *APOB* gene was amplified via polymerase chain reaction (PCR) using 5′-GGAGACTATTCAGAAGCTAA-3′ and 5′-GAAGAGCCTGAAGACTGACT-3′ primers (15 [Bibr B16]). Thermocycling was performed in Biocycler MJ96+/MJ96G Applied Biosystems apparatus (Brazil). Initial DNA denaturation at amplification was obtained with hot start at 80°C for 1 min and 94°C for 2 min, followed by 36 cycles of denaturation at 94°C for 40 s, annealing at 60°C for 45 s, and extension at 72°C for 50 s, followed by a final extension cycle at 72°C for 5 min.

PCR-generated products (amplicons) were digested by *Xba* I (Promega, USA), with a specific recognition site corresponding to TCTAGA at 37°C for 10 h. Each expected restriction pattern (710, 433, and 277bp in T/C subjects) (15) was verified by agarose gel electrophoresis at 2%, with direct visualization analysis under ultraviolet illumination. Samples that generated conflict of interpretation were submitted to additional amplification and enzymatic digestion cycle(s) for confirmation, when necessary.

### Statistical analyses

To test differences between clinical and biochemical measurements according to *APOB* genotypes, statistical analyses compared measures of central tendency by the multivariate analysis of variance (MANOVA) test or the Kruskal-Wallis test for continuous traits with normal or non-normal distribution, respectively. Frequencies were compared with the chi-squared test, including analysis of genotypic frequencies by the Hardy-Weinberg equilibrium. Normal distribution of all variables was assessed using the Kolmogorov-Smirnov test. In addition, when genotypic groups were taken together, Student's *t*-test was used to compare normally distributed continuous variables. For these analyses, P≤0.05 was adopted as the significance level. All analyses were performed using the Statistical Package for Social Sciences software (IBM, USA) for Windows (version 17.0).

## Results

In this study, clinical, biochemical and lifestyle aspects of a sample of 644 individuals assisted in geriatric services were determined. The genotyping of rs693 SNP in the sample revealed that 37.6% were homozygous for the C allele, while 49.2% were heterozygote and the remaining 13.2% corresponded to T homozygotes. The genotypic distribution of this polymorphism was in accordance with the Hardy-Weinberg equilibrium (χ^2^=1.40; df=2; P=0.236).

Inferential analyses according to genotypic groups revealed that our sample of older adults presented a homogeneous pattern regarding basal characteristics such as age and gender (Table 1). Likewise, anthropometric (BMI, body fat), blood pressure (systolic and diastolic blood pressure), metabolic (fasting lipemics and glycemia), and lifestyle variables (caloric and lipid intake) did not differ significantly among groups. Regardless of genotypes, there was a high prevalence of metabolic disorders in the whole sample, with 64% of patients being overweight (BMI >25) and 46% with high body fat percentage (>30% for women and >25% for men) (26,27). Borderline (or slightly supra-physiological) mean levels for traits such as triglycerides, total cholesterol, and systolic blood pressure across all genotypic groups also illustrated this important frequency of metabolic deviations.

**Figure 1:**
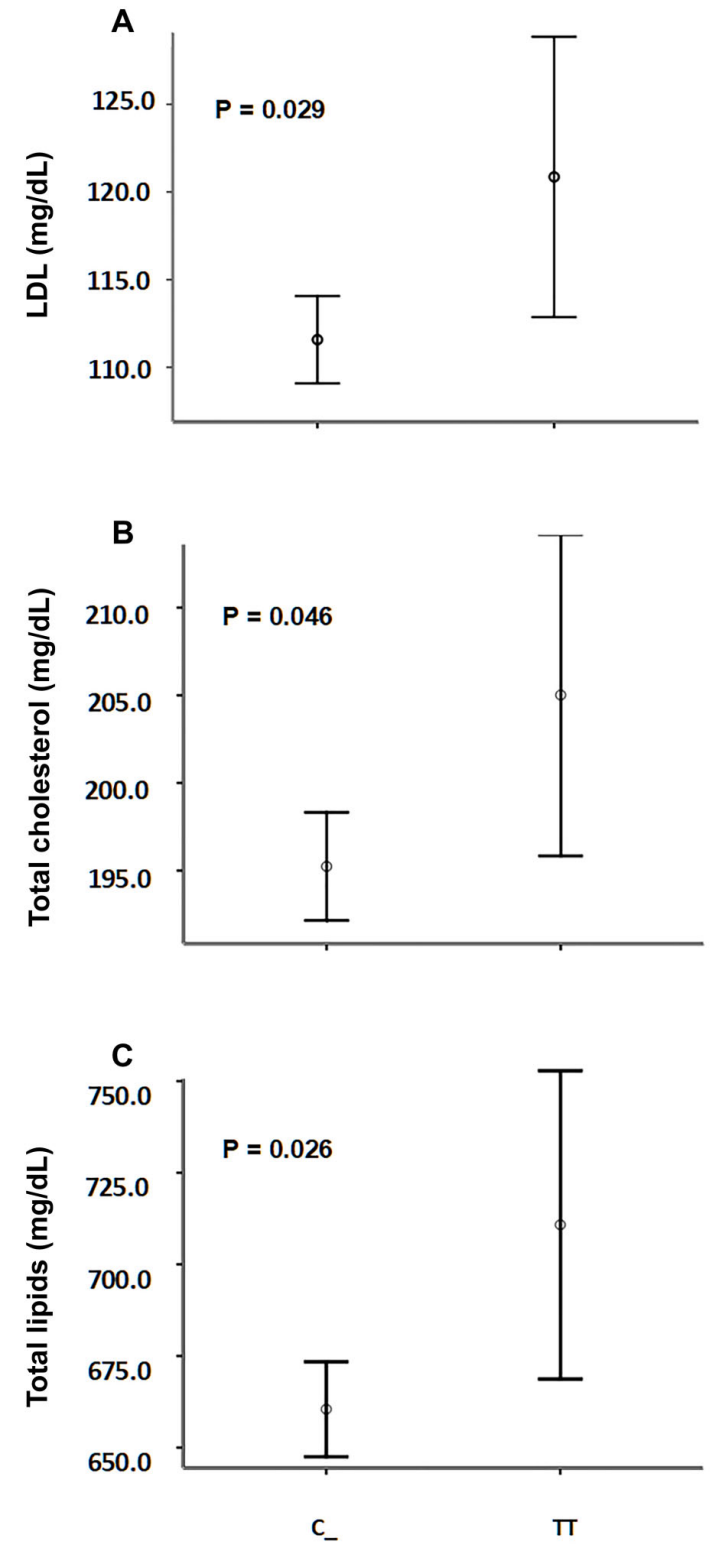
Circulating levels of LDL (**A**), total cholesterol (**B**), and total lipids (**C**) among carriers and non-carriers of the C allele of the apoB rs693 polymorphism. Significance was determined by Student's *t*-test. Vertical bars represent intervals of two standard error deviations.

Among all clinical, biochemical, and lifestyle variables analyzed across genotypes, there was a significant influence of the rs693 allelic variance on levels of LDL cholesterol (P=0.023) and total lipids (P=0.027), as well as a trend on total cholesterol (P=0.061) and serum apoB concentration (Table 1). When considered separately, homozygotes for the T allele had mean serum LDL and total cholesterol levels about 10 mg/dL higher than the respective level observed in heterozygotes and homozygotes for the C allele combined (Figure 1). Complementary analyses according to the Cohen convention (28) allowed categorizing these differences as of small to moderate effect size (*d*) between dichotomized genotypic groups: LDL_TT_ *vs*_C__, *d*=0.35; TC_TT_ *vs*_C__, *d*=0.25. Total lipid levels were about 50 mg/dL higher in carriers of the TT genotype (lipids_TT_ *vs*_C__, *d*=0.41). In this dichotomous arrangement, a decrease in serum apoB among homozygotes for T (83.6±21.9 mg/dL) compared to C carriers (93.5±24.2 mg/dL) remained border line significant (P=0.051), while no other serum or clinical variable was revealed as meaningfully (or marginally) influenced by genotypes.

**Table 1:** Analysis of clinical and biochemical variables of the sample using MANOVA, according to genotypes of the APOB gene.

Variables	*APOB*			P
	(X^-^X^-^) CC (n=242)	(X^+^X^-^) TC (n=317)	(X^+^X^+^) TT (n=85)	
Age (years)	73.8±10.0	73.7±10.4	74.0±10.2	0.970
Gender (% male)	29.8	39.4	35.3	0.101
BMI (kg/m^2^)	27.0±5.0	27.1±4.5	26.4±5.2	0.464
Body fat (%)	28.7±11.8	26.7±11.8	27.8±11.7	0.313
Caloric intake (10^3^ kcal)	2.01±0.48	1.98±0.43	1.94±0.45	0.763
Total lipid intake (%)	35.1±4.7	34.8±4.9	36.0±6.1	0.803
PUFA intake (%)	0.97±0.65	1.01±0.55	0.95±0.67	0.556
MUFA intake (%)	4.32±2.19	3.97±2.02	3.37±2.27	0.288
SFA intake (%)	2.90±1.32	2.88±1.29	2.87±1.37	0.903
Cholesterol intake (mg)	105.5±67.7	103.8±63.6	109.7±71.1	0.772
Serum glucose (mg/dL)	101.5±29.4	104.1±33.0	95.2±22.8	0.123
Systolic BP (mmHg)	140.2±22.9	140.8±23.1	138.2±22.1	0.698
Diastolic BP (mmHg)	80.4±13.0	78.8±13.0	78.6±13.9	0.396
Serum triglycerides (mg/dL)	137.8±69.7	143.2±82.2	140.4±61.3	0.701
Total serum cholesterol (mg/dL)	196.6±35.3	194.2±37.4	205.0±42.0	0.061
LDL cholesterol (mg/dL)	112.9±28.2	110.6±30.3	120.9±36.6^a^	0.023
VLDL cholesterol (mg/dL)	29.1±13.7	28.6±13.6	29.3±12.6	0.861
HDL cholesterol (mg/dL)	53.8±12.6	53.7±12.8	54.4±12.3	0.913
Total serum lipids (mg/dL)	656.0±126.2	660.9±107.8	710.8±145.8^b^	0.027
C-RP (mg/L)	1.4 [0.5; 4.0]	1.4 [0.9; 3.2]	2.3 [0.9; 4.4]	0.213
TSH (μU/mL)	2.0 [1.4; 3.4]	2.1 [1.3; 3.4]	2.2 [1.1; 3.1]	0.727
ApoB (mg/dL)	94.8±25.3	91.1±22.9	83.6±21.9	0.067

Data are reported as means±SD, median [interquartile interval], or ratio within the group. Superscript letters indicate *post hoc* analyses for multiple comparisons using the Scheffé test, adopting P<0.05 when T homozygotes are compared to the TC genotype alone^(a)^ and when compared to all other genotypes combined^(b)^. BMI: body mass index; BP: blood pressure; C-RP: C-reactive protein; HDL: high-density lipoprotein; LDL: low-density lipoprotein; MUFA: monounsaturated fatty acid; PUFA: polyunsaturated fatty acid; SFA: saturated fatty acid intake; TSH: thyroid stimulating hormone; VLDL: very-low-density lipoprotein.

## Discussion

Hyperlipidemia, highlighting elevated LDL cholesterol levels, has been reported to be a contributor to cardiovascular disease. Plasma constituents are determined by a complex interaction between genetics and environment (29), and genetic variations have been closely related to lipid metabolism disorders and atherosclerosis pathogenesis (30). In this sense, one of the genes of particular interest is the *APOB* gene (and its polymorphisms) since its product plays an essential role in the metabolism of circulating lipoprotein particles (3). Considering that the elderly population is largely affected by cardiovascular disorders, the present study investigated the association between the *Xba* I polymorphism (rs693) of the *APOB* and atherosclerotic risk factors (including fasting lipemic profile) of a segment of the Brazilian elderly population.

Our results showed the influence of *Xba* I polymorphism on serum LDL and total cholesterol levels as well as total lipids, with higher mean scores among T homozygotes despite the background scenario of remarkable prevalence of dyslipidemia. Elevated blood levels of these particles are a recognized risk factor for cardiovascular disorders (31). A previous study has found that an increase of 0.7 mM (∼27 mg/dL) in mean total cholesterol levels results in a 25–30% increase in the incidence of coronary diseases and in a 15–20% rise in ischemic stroke events (32). In this sense, our findings tend to bear clinical relevance beyond statistical significance since it modestly (but consistently) associates the *APOB* rs693 SNP with exacerbation of a notoriously atherogenic metabolism.

The literature has investigated the association between rs693 polymorphism and lipemic levels, and our results are in line with studies on the topic as the one from Niu et al. (14), who reported a significant association between the SNP and elevated TC, LDL, and triglycerides levels. Although other studies found divergent results, with no association between the SNP and the lipemic profile of individuals (9,18,19), a meta-analysis supports increased TC, TG, and LDL levels among carriers of the T allele (30). The well-known genetic heterogeneity between populations and a gene-environment interaction may be at least partly responsible for inconsistencies observed in the literature, possibly attributable to intra- and inter-population differences in diet and lifestyle (11). In this aspect, our analyses were monitored for a possible dietary influence from caloric and/or fat intake on lipemic levels of patients, with intakes not varying among genotypes rendering a result unbiased by diet.

Also, clinical evaluations pointed out to a subset (n=88; 13.7%) of the patients as active consumers of lipid-lowering drugs. However, we do not believe that our inferential analyses was severely influenced by the consumption of this class of drugs since the chi-squared test did not reveal quantitative variations in the distribution of users and non-users of this class of medication among any tested genotypic configurations (P>0.05), assembled or not. An influence from prior cardiovascular conditions was also not observed. It is worth noticing that part of our sample was constituted by patients eligible for secondary prevention. Although existent, the clinically diagnosed cases of coronary heart disease (n=65; 10.0%), congestive heart failure (n=48; 7.4%), prior myocardial infarction (n=43; 6.6%), and arrhythmia (n=29; 4.5%) (to name the more prevalent) were equally distributed across genotypes, and no differential lipemic level was observed in any of these subsets or in the whole subset of CVD-carrying older adults (P>0.05) compared to those with no clinically diagnosed cardiovascular condition. In addition, the sample size provided substantial statistical power for association estimates.

The mechanism behind the association between the *Xba* I polymorphism and lipemic levels is not well defined, since SNP does not cause changes in the amino acid sequence. It is presumed that this polymorphism is in linkage disequilibrium with functional mutations in the *APOB* loci or neighboring sequences, implicating in alterations of structure or expression and affecting lipemic levels as observed herein and elsewhere (10). This silent variation is only 600 and 900 base pairs away from the two coding domains of the LDL receptor binding site and has been proposed to be in haplotypic configuration with allelic variations affecting these regions (9).

Saha et al. (33) showed strong linkage between the *Xba* I SNP and an Ins/Del polymorphism of the *APOB* gene, whose Del variant of the latter is also consistently associated with serum LDL elevations (34). In a Brazilian sample, the haplotype formed by the *Del* and T alleles showed strong association with a mixed lipemic profile of higher risk for coronary artery diseases (35). The actual effect of the *Del* allele on serum lipid levels (and resulting vascular risk) is also not fully understood. It has been proposed that the three amino acids (Leu-Ala-Leu) missing in the *Del* variant of *APOB* can alter the hydrophobicity of the signal peptide and impair translocation of the newly synthesized apoB from the cytoplasm to the endoplasmic reticulum, affecting the secretion of apoB as VLDL from hepatocytes (36). LDL and TC elevations in this context would justifiably be augmented since deficient assembly of VLDL implies direct LDL production and secretion by the liver as a compensatory mechanism (37). In addition, deficient assembly of VLDL can determine hepatic cholesterol retention and promote endogenous apoB breakdown (38) to the point of justifying the trend for reduced apoB levels observed herein. However, the absence of an association between *Xba* I SNP and VLDL in our study does not corroborate these arguments. Nonetheless, the effects of the Ins/Del polymorphism on the circulating LDL profile have already been reported in the absence of a significant effect on non-LDL plasma cholesterol forms (39), indicating that the changes promoted by this haplotype may reverberate mostly on the apo-content within lipoproteins rather than on the actual serum concentrations of these particles (40).

Despite the standardization of participants in terms of cognitive performance and the presence of classic risk factors for vascular disorders, the present study has limitations. Despite the effect of exercise on lipemics, the study did not evaluate levels of physical fitness among subjects. In addition, neither the remarkable genetic admixture of the Brazilian population nor unusual dietary habits were considered or controlled for in our analyses.

In summary, our results corroborated that genetic variations in apolipoprotein B affected the lipemic profile of Brazilian elderly individuals living in urban conditions and may contribute to atherosclerosis. Given the heterogeneity in the literature, and considering that vascular disorders result from complex interactions between genes and environment, we suggest further studies to identify the physiological impact of allelic variations of *APOB* (including the *Xba* I polymorphism and others with strong effect) considered in the light of environmental factors with which they interact.

## References

[1] WHO (World Health Organization), World report on ageing and health (2015). Geneva: World Health Organization.

[2] WHO (World Health Organization) (2018). World health statistics 2018: monitoring health for the SDGs, Sustainable Development Goals.

[3] Daheshpour MS, Faam B, Hedayati M, Eshraghi P, Azizi F (2011). ApoB (*Xba* I) polymorphism and lipid variation in Teharnian population. Eur J Lipid Sci Technol.

[4] Kodogo V, Zhou DT, Ocktedalen O, Duri K, Stray-Pedersen B, Gomo E (2016). Apolipoprotein B gene polymorphisms and dyslipidemia in HIV infected adult Zimbabweans. Open AIDS J.

[5] Hassan NE, El-Masry SA, Zarouk WA, Elneam AIA, Rasheed EA, Mahmoud MM (2015). Apolipoprotein B polymorphism distribution among a sample of obese Egyptian females with visceral obesity and its influence on lipid profile. J Genet Eng Biotechnol.

[6] Wang YT, Li Y, Ma YT, Yang YN, Ma X, Li XM (2018). Association between apolipoprotein B genetic polymorphism and the risk of calcific aortic stenosis in Chinese subjects, in Xinjiang, China. Lipids Health Dis.

[7] Benn M (2009). Apolipoprotein B levels, APOB alleles, and risk of ischemic cardiovascular disease in the general population, a review. Atherosclerosis.

[8] Sotos-Prieto M, Peãalvo JL (2013). Genetic variation of apolipoproteins, diet and other environmental interactions; an updated review. Nutr Hosp.

[9] Casillas-Muãoz F, Valle Y, Muãoz-Valle JF, Martínez-Fernandez DE, Reynoso-Villalpando GL, Flores-Salinas HE (2018). APOA1 and APOB polymorphisms and apolipoprotein concentrations as biomarkers of risk in acute coronary syndrome: Relationship with lipid-lowering therapy effectiveness. Med Clin.

[10] Xiao D, Huang K, Chen Q, Huang B, Liu W, Peng Y (2015). Four Apolipoprotein B gene polymorphisms and the risk for coronary artery disease: a meta-analysis of 47 studies. Genes Genom.

[11] Srivastava N, Prakash J, Srivastava A, Agarwal CG, Pant DC, Mittal B (2013). Association of apolipoprotein B XbaI gene polymorphism and lipid profile in northern Indian obese. Indian J Hum Genet.

[12] Rodrigues AC, Sobrino B, Genvigir FD, Willrich MA, Arazi SS, Dorea EL (2013). Genetic variants in genes related to lipid metabolism and atherosclerosis, dyslipidemia and atorvastatin response. Clin Chim Acta.

[13] Nikolajevic Starcevic J, Santl Letonja M, Praznikar ZJ, Makuc J, Vujkovac AC, Petrovic D (2014). Polymorphisms XbaI (rs693) and EcoRI (rs1042031) of the ApoB gene are associated with carotid plaques but not with carotid intima-media thickness in patients with diabetes mellitus type 2. Vasa.

[14] Niu C, Luo Z, Yu L, Yang Y, Chen Y, Luo X (2017). Associations of the APOB rs693 and rs17240441 polymorphisms with plasma APOB and lipid levels: a meta-analysis. Lipids Health Dis.

[15] Liu YL, Zhang YB, Li Y, Ma RL, Cai WW, Lin-Jiang L (2014). Correlation between the Xba I polymorphism of apoB gene and serum lipid profiles in Li ethnic group. Asian Pac J Trop Med.

[B16] Tsunoda K, Harihara S, Tanabe Y, Dashnyam B (2012). Polymorphism of the apolipoprotein B gene and association with plasma lipid and lipoprotein levels in the Mongolian Buryat. Biochem Genet.

[17] Liu FL, Lu WB, Niu WX (2010). XbaI polymorphisms of apolipoprotein B gene: another risk factor of gallstone formation after radical gastrectomy. World J Gastroenterol.

[18] Bogari NM, Abdel-Latif AM, Hassan MA, Ramadan A, Fawzy A (2015). No association of apolipoprotein B gene polymorphism and blood lipids in obese Egyptian subjects. J Negat Results Biomed.

[19] Whitley E, Ball J (2002). Statistics review 4: Sample size calculations. Crit Care.

[20] Sociedade Brasileira de Cardiologia, Sociedade Brasileira de Hipertensão, Sociedade Brasileira de Nefrologia (2010). VI Brazilian Guidelines on Hypertension. Arq Bras de Cardiol.

[21] Friedewald WT, Levy RI, Friedrickson DS (1972). Estimation of the concentration of low-density lipoprotein cholesterol in plasma, without use of the preparative ultracentrifuge. Clin Chem.

[22] Folstein MF, Folstein SE, Mchugh PR (1975). “Mini-mental state” A practical method for grading the cognitive state of patients for the clinician.. J Psychiatr Res.

[23] Castro-Costa E, Fuzikawa C, Uchoa E, Firmo JO, Lima-Costa MF (2008). Norms for the mini-mental state examination: adjustment of the cut-off point in population-based studies (evidences from the Bambuí Health Aging Study). Arq Neuro-Psiquiatr.

[24] Philippi ST (2002). Tabela de composição de alimentos: suporte para decisão nutricional. 2th. ed..

[25] Paula RS, Souza VC, Toledo JO, Ferreira AP, Brito CJ, Gomes L (2016). Habitual dietary intake and mediators of the inflammaging process in Brazilian older women. Aging Clin Exp Res.

[26] World Health Organization (1995). Physical status: the use and interpretation of anthropometry.

[27] Position of the American Dietetic Association and the Canadian Dietetic Association: nutrition for physical fitness and athletic performance for adults (1993). J Am Diet Assoc.

[28] Cohen J (1992). A power primer. Psychol Bull.

[29] Paula RS, Souza VC, Benedet AL, Souza ER, Toledo JO, Moraes CF (2010). Dietary fat and apolipoprotein genotypes modulate plasma lipoprotein levels in Brazilian elderly women. Mol Cell Biochem.

[30] Gu W, Zhang M, Wen S (2015). Association between the APOB XbaI and EcoRI polymorphisms and lipids in Chinese: a meta-analysis. Lipids Health Dis.

[31] Duncan MS, Vasan RS, Xanthakis V (2019). Trajectories of blood lipid concentrations over the adult life course and risk of cardiovascular disease and all-cause mortality: observations from the Framingham study over 35 years. J Am Heart Assoc.

[32] Zhang X, Patel A, Horibe H, Wu Z, Barzi F, Rodgers A (2003). Cholesterol, coronary heart disease, and stroke in the Asia Pacific region. Int J Epidemiol.

[33] Saha N, Tay JS, Heng CK, Humphries SE (1993). DNA polymorphisms of the apolipoprotein B gene are associated with obesity and serum lipids in healthy Indians in Singapore. Clin Genet.

[34] Zhang JZ, Zheng YY, Yang YN, Li XM, Fu ZY, Dai CF (2015). Association between apolipoprotein B gene polymorphisms and the risk of coronary heart disease (CHD): an update meta-analysis. J Renin Angiotensin Aldosterone Syst.

[35] Machado MO, Hirata MH, Bertolami MC, Hirata RD (2001). Apo B gene haplotype is associated with lipid profile of higher risk for coronary heart disease in caucasian brazilian men. J Clin Lab Anal.

[36] Benhizia F, Ginsberg HN, Humphries SE, Talmud PJ (2001). Variation in the human apoB signal peptide modulates apoB17 translocation. Biochem Biophys Res Commun.

[37] Plonné D, Schulze HP, Kahlert U, Meltke K, Seidolt H, Bennett AJ (2001). Postnatal development of hepatocellular apolipoprotein B assembly and secretion in the rat. J Lipid Res.

[38] Liao W, Hui TY, Young SG, Davis RA (2003). Blocking microsomal triglyceride transfer protein interferes with apoB secretion without causing retention or stress in the ER. J Lipid Res.

[39] Kallel A, Feki M, Elasmi M, Souissi M, Sanhaji H, Omar S (2007). Apolipoprotein B signal peptide polymorphism: distribution and influence on lipid parameters in Tunisian population. Physiol Res.

[40] Chen Y, Lin M, Liang Y, Zhang N, Rao S (2016). Association between apolipoprotein b XbaI polymorphism and coronary heart disease in Han Chinese population: a meta-analysis. Genet Test Mol Biomarkers.

